# 3-Year Clinical Performance of a New Pit and Fissure Sealant

**DOI:** 10.3390/jcm11133741

**Published:** 2022-06-28

**Authors:** Helen Schill, Peter Gräser, Katharina Bücher, Jan Pfisterer, Yeganeh Khazaei, Lukas Enggist, Reinhard Hickel, Jan Kühnisch

**Affiliations:** 1 Department of Conservative Dentistry and Periodontology, School of Dentistry, Ludwig-Maximilians-Universität München, 80336 Munich, Germany; hschill@dent.med.uni-muenchen.de (H.S.); kbuecher@dent.uni-muenchen.de (K.B.); pfisterer@dent.med.uni-muenchen.de (J.P.); hickel@dent.med.uni-muenchen.de (R.H.); 2 Dental Practice, 8820 Wädenswil, Switzerland; pg@praxis-graeser.ch; 3 Statistical Consultation Laboratory (StaBLab), Faculty of Mathematics, Informatics and Statistics, Ludwig-Maximilians-University, 80799 Munich, Germany; yeganekhazaei@gmail.com; 4 Ivoclar Vivadent AG, FL-9494 Schaan, Liechtenstein; lukas.enggist@ivoclar.com

**Keywords:** dental caries, detection and diagnosis, prevention, pit and fissure sealant, retention rate, survival probability, split-mouth design, RCT, Kaplan–Meier statistics

## Abstract

The aim of this 3-year, randomized clinical trial (RCT) in split-mouth design was to explore the clinical survival of a Bis-GMA-free pit and fissure sealant (Helioseal F Plus) in comparison to a control material (Helioseal F). The initial population consisted of 92 adolescents. Follow-ups took place after one year (N = 85), two years (N = 82) and three years (N = 76) after application. At each examination, sealant retention and the presence of caries were recorded. The statistical analysis included the calculation of Kaplan–Meier survival curves, log-rank tests and a Cox proportional hazard regression model. No adverse events were documented. The proportion of completely intact sealants and those with minimal loss was almost identical in both groups, at 84.3% (Helioseal F; 113/134) and 81.7% (Helioseal F Plus; 107/131) after three years of observation. The regression analysis revealed an operator dependency, but no significant differences were found between the materials, the study centers, the chosen isolation technique, patient age or sex. After 3 years, 91.7% and 100.0% of all molars were free of non-cavitated carious lesions or carious cavities, respectively. It can be concluded that the new fissure sealing material can be considered as at least equivalent in terms of survival and retention behavior compared to the predecessor material.

## 1. Introduction

Pit and fissure sealing is a widely accepted, evidence-based preventive dental measure used on caries-prone pits and fissures, especially in young permanent molars, with the aim of preventing the onset of new caries or arresting existing non-cavitated caries lesions [1]. The efficacy of sealants has been researched and validated in several clinical investigations, systematic reviews and meta-analyses [2,3,4,5]. Today, the most-used sealants are methacrylate-based, white opaque, flowable and light-curing, enabling their safe, fast and effective application in the main target group, children and adolescents. In terms of composition, sealants have been fully-developed established products for decades, and have not undergone any substantial changes. Aimed at reducing potential toxicity, interest surrounding a Bisphenol A glycidyl methacrylate (Bis-GMA)-free dental material has, however, increased in recent years [6,7,8,9,10,11,12].

A Bis-GMA-free fissure sealant, Helioseal F Plus (Ivoclar Vivadent AG, Schaan, Liechtenstein), was introduced to the dental market recently. A two-year randomized clinical trial (RCT) showed that Helioseal F Plus (Ivoclar Vivadent AG, Schaan, Liechtenstein) exhibited similar clinical performance to its predecessor product, Helioseal F (Ivoclar Vivadent AG, Schaan, Liechtenstein), and could therefore be recommended for clinical use [13]. To provide more long-term clinical data, the existing two-year RCT was extended by another year. The aim of the present study was to report the three-year follow-up data for the Bis-GMA-free test sealant (Helioseal F Plus, Ivoclar Vivadent AG, Schaan, Liechtenstein) in comparison to the control product (Helioseal F, Ivoclar Vivadent AG, Schaan, Liechtenstein). The null hypothesis proposed that there were still no statistically significant differences between the test and control materials compared to the two-year published results.

## 2. Materials and Methods

### 2.1. Study Design, Ethical Approval & Sample Size Calculation

The original split-mouth-designed in vivo study was envisioned as a prospective, two-center RCT to compare the test sealant (Helioseal F Plus, Ivoclar Vivadent AG, Schaan, Liechtenstein) with the control material (Helioseal F, Ivoclar Vivadent AG, Schaan, Liechtenstein) [13]. The clinical trial as well as the extension received ethical approval from the corresponding ethical boards (Ludwig-Maximilians-University of Munich: Project number 18–319; Cantonal Ethics Committee in Zurich: Basec-Number 2018-00707). The Department of Conservative Dentistry and Periodontology of the LMU Munich, Germany, and a dental practice in Wädenswil, Switzerland, managed the clinical treatments and observations between September 2018 and March 2022. The ethical standards of the institutional research board, the modified Helsinki Declaration and the CONSORT guidelines served as the foundation for the implementation of the RCT [13,14,15,16].

A sample size calculation was performed using G-Power software version 3.1.9.7. [17], beforehand. Here, an alpha of 5%, a confidence interval of 95% and an effect size of 0.60 with two groups, each with at least 40 individuals, yielded a power of 0.80 [13]. The study population was recruited from the patient pool in each study center.

### 2.2. Inclusion Criteria & Follow-Ups

Children and adolescents with an ASA status >1 aged between 5 and 18 years were included. Patients had either an increased caries risk, healthy teeth but with fissures susceptible to caries, or fissures with an initial lesion limited to the enamel [1,12,18,19]. Known allergies or intolerances to methacrylates or other substances of fissure sealants or any other restorative filling materials were considered exclusion criteria [20,21]. To be included, at least one fully or partially unsealed pair of permanent first or second inferior or superior molars had to show an ICDAS score <3 [22] and no hypomineralization or aberrations [13]. If the inclusion criteria were fulfilled and both the (fully informed) patients and their guardians had agreed to participate in the study after extensive information, the fissure sealants were placed by one of the responsible dentists (LMU: JK; HS Wädenswil: PG) in a separate appointment. The follow-up examinations were arranged after one month (7–28 days after sealant application), six months (±4 weeks), one year (±2 months), two years (±2 months) and three years (±6 months). Patients who could not attend follow-up appointments were contacted up to two times before being categorized as ‘lost to follow-up’. A patient’s or legal guardian’s wish to leave the study or the complete loss or partial loss of a sealant with initial caries lesions and necessary resealing led to drop-out [13].

### 2.3. Blinding and Randomization

To ensure an independent assessment of sealant quality, blinding between the operator and evaluator was set up. This was only achievable at LMU. Sealants were photographed at each visit to allow the study team to conduct an independent sealant evaluation. A double-blind study design was possible at the LMU, while a single-blind study design was possible at the dental practice. The operator opened one of the consecutively numbered and sealed envelopes containing the material in question shortly before sealing. Neither the patient nor the study team were informed about the material up to this point [13].

### 2.4. Study Materials

The test material (Helioseal F Plus, Ivoclar Vivadent AG, Schaan, Liechtenstein, LOT at LMU and at the practice: W96091) is a newly formulated methacrylate-based hydroxy-methyl methacrylate (HEMA) phosphate, aromatic aliphatic urethane-dimethacrylate (UDMA), white-pigmented (silicon dioxide, titanium oxide), fluoride-releasing (aluminium fluorosilicate glass) and light-curing (campherquinone with absorption at a 400–500 nm wavelength) fissure sealant. According to the manufacturer, the test material not only claims to be Bis-GMA-free, but also exhibits more thixotropic behavior, enhances flow into deep fissures and has a short light curing time of 10 s. Helioseal F (Ivoclar Vivadent AG, Schaan, Liechtenstein, LOT at LMU and at the practice: X23069) was chosen as the control material. The monomer matrix consists of Bis-GMA, urethane dimethacrylate and triethylene glycol dimethacrylate. The fillers are highly dispersed silicon dioxide and fluorosilicate glass.

### 2.5. Sealant Application

Prior to the sealant application all trained dentists (LMU: JK & HS, dental practice: PG) conducted a professional tooth cleaning. At LMU, all surfaces were cleaned with a medium abrasive fluoride-free polishing paste (Proxyt^®^ Medium Prophy Paste, RDA 36 Ivoclar Vivadent AG, Schaan, Liechtenstein, LOT: X19757) and a rotating brush (Prophy Brush, Hager & Werken GmbH & Co KG, Duisburg, Germany). At the dental office in Wädenswil, all teeth were cleaned with a water-infused air powder polishing system (Prophy Mate Neo and Flash Pearl, NSK Europe, Bensheim, Germany).

Following the manufacturer’s instructions, both materials were placed on the cleaned teeth, either in relative isolation (University) or with absolute isolation with rubber dam (Dental office). Afterwards, an etching procedure with 37% phosphoric acid gel (Total Etch^®^, Ivoclar Vivadent AG, Schaan, Liechtenstein, LOT: X23292) was carried out for 60 s. The tooth surface was then rinsed with water and air-dried until a chalky-white enamel surface was visible. The spread sealant was distributed either with the cannula and dental probe (dental office) or brush stick (University: Microbrush^®^, Microbrush Int., Grafton, WI, USA) to reach all pits and fissures without incorporating air bubbles. The test and control materials were exposed for 10 s (Helioseal F Plus) and for 20 s (Helioseal F) with an LED curing lamp with a light intensity of 1.200 mW/cm^2^ ±10% (Bluephase Style^®^, Ivoclar Vivadent AG, Schaan, Liechtenstein), respectively. The oxygen-inhibition layer was removed by using the bristle brush again. If necessary, the occlusion was corrected with a polishing cup (OptraPol^®^ Small Flame, Ivoclar Vivadent AG, Schaan, Liechtenstein). Finally, fluoride varnish was applied (University: Fluor Protector or Fluor Protector S, Ivoclar Vivadent AG, Schaan, Liechtenstein; Dental office: Elmex^®^ fluid, CP GABA, Hamburg, Germany) [13].

### 2.6. Dental Examinations and the Calibration of the Study Team

The dental status included the documentation of non-cavitated and cavitated caries lesions, as well as dental restorations, in accordance with recognized standards [23,24,25,26]. Sealants and the extent of their retention were categorized into the following categories [27,28,29]: occlusal surfaces without a sealant (Score 0), occlusal surfaces with a fully intact fissure sealant (sufficient, Score 1), intact sealant with minor loss of the material up to one-third in the periphery of the fissure pattern (sufficient, Score 2), occlusal surface with retention of the material in the main fissure but loss of the material exceeding one-third of the fissure pattern (insufficient, Score 3) and near complete loss of the material and re-exposure of the main fissures (insufficient, Score 4). Discoloration was divided into two categories: no discoloration documented (Score 1) and discoloration present (with sub-variables if polishable or not, Score 0). Marginal integrity was counted either as complete integrity to the surface and therefore sufficient (Score 1), or partial or incomplete and therefore insufficient (Score 0) [13].

To standardize examinations and test methods, the two operators (HS, PG) and examiners (KB, JP, PG) were trained in a two-day theoretical and practical calibration training by a specialized dentist (JK). Intra- and inter-examiner reproducibility was measured for all examiners and indices used. The weighted Kappa values for the intra- and inter-examiner reproducibility of the study team were good to excellent (ICDAS/UniViSS criteria: 0.90–0.97 (intra) and 0.89–0.97 (inter); DMF index: 0.85–0.86 (intra) and 0.76–0.92 (inter); sealant retention: 0.91–0.94 (intra) and 0.92–0.97 (inter)). The reproducibility was rated as excellent in accordance with Fleiss [30].

### 2.7. Statistical Analysis

The anonymized data was collected by using a validated data entry and management system (“Evaluation”, Ivoclar Vivadent AG, Schaan, Liechtenstein). Only the principal investigator was granted access to the data set by entering an access code. As a result, the anonymity of the data regarding third parties and the sponsor was guaranteed. The raw data were checked for plausibility after export and archived in anonymized form at both study sites, where it will be retained for 10 years. Excel spreadsheets were used to complete descriptive analysis (Microsoft Excel for Office 365, Version 16.60, Microsoft Corporation, Redmond, WA, USA). R software was used for exploratory statistical analysis (version R-4.1.1 R Development Core Team, Vienna, Austria & survival analysis package; version 3.3-1, https://cran.r-project.org, accessed on 19 April 2022). With a 95% confidence interval, the significance threshold was chosen at = 0.05. Data on survival probability was generated using Kaplan–Meier estimators [31,32]. Log-rank tests were used to analyze differences in survival rates. Using a Cox proportional hazard regression analysis [33], the effect of variables such as age, sex, study center, operator and material on sealant survival after following follow-ups and an overall assessment was examined.

## 3. Results

Of 92 individuals who were initially included in the prospectively designed split-mouth study, 76 adolescents (mean age 12.7 years, standard deviation 2.7 years) could be followed up within the scheduled interval after three years (Table 1). This equates to an overall attrition rate of 17.4%.

After three years, a total of 265 sealants could be rechecked. At the LMU study center, a total of 13 sealants (Table 1) were excluded from further statistical evaluation because they had been resealed in the meantime as part of preventive care. Importantly, no adverse events were documented throughout the study period.

Data regarding sealant retention are summarized in detail in Table 2. The proportion of completely intact sealants at the three-year recall was almost identical in both groups, at 62.7% (Helioseal F; 84/134) and 61.8% (Helioseal F Plus; 81/131). Retention losses occurred equally in both material groups: Helioseal F = 50/134 (37.3%) and Helioseal F plus = 50/131 (38.2%). Most of the retention losses were classified as minimal (all sealants = 55/265 (20.8%); Helioseal F = 29/134 (21.6%), Helioseal F plus = 26/131 (19.9%)). In contrast, 45 sealants (17.0% of all sealants) were affected by more extensive retention loss: central retention (N = 27) and almost complete loss (N = 18). These descriptive numbers were found in approximately equal proportions in both material groups (Table 2). If the retention data are assessed according to the internationally accepted criteria for evaluating the success of fissure sealants—summary of intact sealants and minor retention losses as treatment success—the proportion of sufficient sealants amounted to 84.3% (Helioseal F; 113/134) and 81.7% (Helioseal F Plus; 107/131).

Marginal discoloration was present in a total of 15/134 Helioseal F sealants (11.2%) and 9/131 Helioseal F Plus sealants (6.8%) after three years (Table 2). The study center in Switzerland recorded more marginal discoloration compared to the LMU study center. Marginal integrity loss was not documented after three years (Table 2). The case recorded previously was resealed in the meantime. Overall, the frequency of this feature is therefore negligible.

After three years, 91.7% and 100.0% of all molars were free of non-cavitated carious lesions or carious cavities, respectively. With respect to the caries-preventive effect, it remains to be stated that in 8.3% of all cases (22/365) the appearance of caries-related discolorations (in the sense of non-cavitated caries lesions) was observed after three years; cavitations were not registered in any case (Table 2). It should be added that the occurrence was independent of the material and the cases were evenly distributed in the Helioseal F Plus (N = 11) and Helioseal F (N = 11) groups. No differences between upper and lower molars were observed.

**Figure 1:**
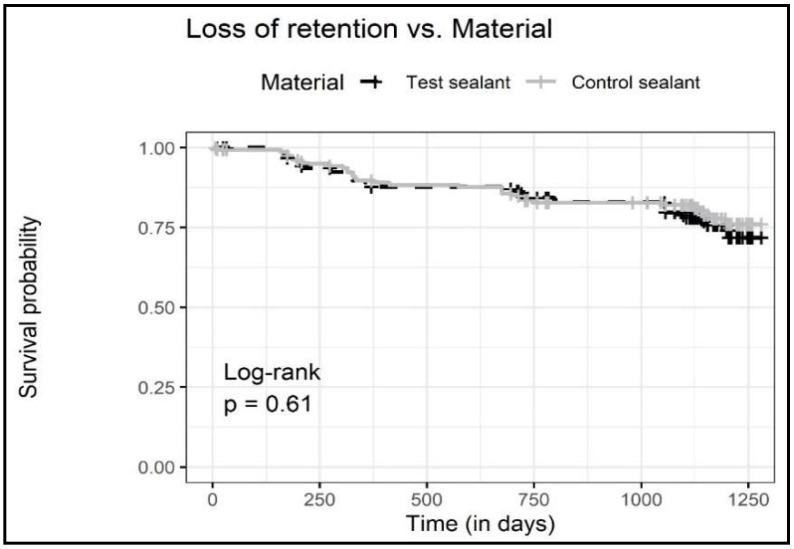
Kaplan–Meier survival curves for both tested sealant materials.

The dataset was further explored by computing Kaplan–Meier survival curves (Figure 1) and by applying a logistic regression model (Table 3). In detail, the survival curves for both materials were found to be near to identical after three years observation time, and the corresponding log-rank test (*p* = 0.61) indicated an insignificant difference (Figure 1). The Cox proportional hazard regression analysis including the cumulative retention data as well as important co-variables indicated only an operator dependency (Table 3). Furthermore, no significant differences regarding age, sex, study center or sealant material were detectable (Table 3).

## 4. Discussion

This three-year RCT demonstrated no difference in the clinical performance between the novel Bis-GMA-free sealant material and the corresponding control product. Therefore, the initially formulated null hypothesis was accepted. Even though the topic is not novel and fissure sealants have not undergone substantial changes, adding more information and data by extending the follow-up period contributes to the investigation. The separate presentation of two- and three-year data follows the recommendation for controlled clinical studies in dental restorative materials [34]. It appears to be of practical relevance because the novel Bis-GMA-free material behaved similarly to the control material, but with possible lower toxicity and additional advantageous material properties. As a result, it may be deemed more beneficial in daily routines.

If the proportion of available three-year recalls is extrapolated to the primarily included number of participants, then 82.6% (76/92) of all patients and 80.8% of all sealants (265/328) were clinically followed up despite pandemic-related limitations. It should be mentioned that 13 teeth received a resealing between the two- and three-year visits and were excluded from the statistical analysis. A resealing was mostly undertaken due to retention losses in caries risk patients. However, the exclusion of this small group means that the clinical parameters of retention, marginal integrity, marginal discoloration and caries formation are somewhat more favorable. Therefore, this should be considered from a methodological point of view when interpreting the clinical performance data.

When considering the longevity of sealants, the material retention is the most frequently reported parameter. The rates in terms of sufficiently sealed occlusal surfaces amounted to 81.7% (Helioseal F Plus) and 84.3% (Helioseal F). Both values are similar and in line with the estimated expectations from previously conducted clinical trials and meta-analyses. Furthermore, the data align with other studies on retention behavior with estimates of around 10% retention loss per year of observation [2,3].

Another important aspect concerns caries development on the sealed teeth. After three years, 91.7% and 100.0% of all molars were free of non-cavitated carious lesions or carious cavities after three years, respectively. If the group of non-cavitated carious lesions (8.3% of all molars; 22/365) is considered, this proportion increased especially in the last year of observation. While only 5 non-cavitated carious lesions were documented at the two-year recall, there were 22 cases after three years. The following aspects should be discussed. Even if the caries incidence in the study group was already assessed as low, there are caries risk patients who gradually develop new caries lesions in childhood and adolescence depending on their individual behavior. This is equally possible on occlusal surfaces when fissures and pits were not completely sealed. This ultimately underlines the importance of continually monitoring sealants. As caries development is multi-factorial, depending on more than just a sealant, there are no clear statements on the extent to which sealant fluoride release inhibits caries development or remineralizes within the fissure [11,35].

When plotting the retention data as Kaplan–Meier survival curves, no differences became obvious (Figure 1). The Cox regression model confirmed this premise and detected no significant influence for patient age, sex, study center or sealant material (Table 3). Only an operator dependency was observed for the practitioner with less clinical experience in managing children and adolescents (~5 years) compared to the other two practitioners (>20 years). Although no significant differences were observed between study centers, there could be some interactions between the clinical experience of practitioners and isolation methods (cotton roll vs. rubber dam). It could be discussed that the combination of relative isolation with cotton roll with a less experienced practitioner could significantly reduce the retention. Additionally, the retention rate could be increased with expanding experience and rubber dam isolation. This highlights that soft skills in pediatric dentistry, e.g., communication, behavior management and a smooth teamwork flow, may be underestimated but affect data quality in other study setups as well.

This research has both strengths and limitations. The study’s RCT design and adherence to the CONSORT reporting standards should be considered as strengths [15,16,36]. To preserve patient- and evaluator-independent scoring, photographs were obtained at each time point, allowing controversial events to be reviewed subsequently with the principal investigator or by the research group. Nonetheless, the variation in blinding methods between the two research facilities should be noted. As the dental practitioner had to evaluate his own fissure sealants, the data gathered could also be biased. Differences in the preparation and isolation prior to applying the fissure sealant should also not be disregarded. The rate of intact fissure sealants may have been raised if the role of absolute isolation (rubber dam) in restorative materials was also applied to fissure sealants [37]. In this RCT, no statistically significant difference between study centers was found regarding retention. Neither cleaning with a rotating bristle and cotton roll isolation (LMU) or water-infused air-powder polishing and rubber dam (Dental practice) affected the sealant survival. Therefore, the workflows used can be considered equivalent [38,39,40].

## 5. Conclusions

This three-year RCT exhibited high sealant retention rates and a similar clinical performance for the new Bis-GMA-free material in comparison to the predecessor product. The proportion of completely intact sealants and those with minimal loss was almost identical in both groups, at 84.3% (Helioseal F; 113/134) and 81.7% (Helioseal F Plus; 107/131) after three years of observation. The test sealant can therefore be recommended for clinical use.

## Figures and Tables

**Table 1:** Overview of Study population. ^a^ Drop-outs from previous examinations must be taken into account when calculating the respective total number. One case cannot be evaluated due to orthodontic treatment.

	Baseline	1 Year	2 Year	3 Year
N Patients	92	85 ^a^	82 ^a^	76 ^a^
Female/Male	51/41	47/38	45/37	41/35
LMU University (Germany)	51	48	47	41
Praxis Wädenswil (Swiss)	41	37	35	35
N Patients (No Show)	-	3	6	12
N Patients (Drop Out)	-	4	-	-
N Sealants	328	305 ^a^	297	265
Helioseal F	164	152	148	134
Resealing	-	-	-	5
Helioseal F plus	164	153 ^a^	149	131
Resealing	-	-	-	8

**Table 2:** Retention rates, marginal integrity and new caries lesions for both sealant materials at baseline examination (BL), 1-, 2- and 3-year follow-up. Odd number due to one non-evaluable case after one year.

	University								Dental Practice							
	Test Sealants				Control Sealants				Test Sealants				Control Sealants			
Sealant Retention	BL	1 Year	2 Year	3 Year	BL	1 Year	2 Year	3 Year	BL	1 Year	2 Year	3 Year	BL	1 Year	2 Year	3 Year
Intact sealant	83	51	41	21	83	49	44	30	81	65	60	60	81	62	56	54
Minimal loss of retention	0	12	18	19	0	13	16	17	0	9	9	7	0	11	12	12
Main retention complete	0	6	10	11	0	12	11	12	0	0	1	1	0	1	3	3
Nearly complete sealant loss	0	8	8	10	0	2	6	5	0	0	0	2	0	0	0	1
Complete sealant loss	0	2	1	0	0	2	0	0	0	0	1	0	0	0	0	0
Total	83	79	78	61	83	78	77	64	81	74	71	70	81	74	71	70
**Marginal Discoloration**	**BL**	**1 year**	**2 year**	**3 year**	**BL**	**1 year**	**2 year**	**3 year**	**BL**	**1 year**	**2 year**	**3 year**	**BL**	**1 year**	**2 year**	**3 year**
None	83	79	78	61	83	78	77	63	81	71	68	61	81	74	68	56
Present	0	0	0	0	0	0	0	0	0	3	3	9	0	0	3	14
Total	83	79	78	61	83	78	77	64	81	74	71	70	81	74	71	70
**Marginal Integrity**	**BL**	**1 year**	**2 year**	**3 year**	**BL**	**1 year**	**2 year**	**3 year**	**BL**	**1 year**	**2 year**	**3 year**	**BL**	**1 year**	**2 year**	**3 year**
Sufficient	83	78	78	61	83	77	76	64	81	74	71	70	81	74	71	70
Insufficient	0	1	0	0	0	1	1	0	0	0	0	0	0	0	0	0
Total	83	79	78	61	83	78	77	64	81	74	71	70	81	74	71	70
**New Caries Lesions on Sealed Teeth**	**BL**	**1 year**	**2 year**	**3 year**	**BL**	**1 year**	**2 year**	**3 year**	**BL**	**1 year**	**2 year**	**3 year**	**BL**	**1 year**	**2 year**	**3 year**
No caries	83	77	75	50	83	77	75	53	81	74	71	70	81	74	71	70
Non-cavitated caries	0	2	3	11	0	1	2	11	0	0	0	0	0	0	0	0
Cavitated caries	0	0	0	0	0	0	0	0	0	0	0	0	0	0	0	0
Total	83	79	78	61	83	78	77	64	81	74	71	70	81	74	71	70

**Table 3:** Results from the Cox hazard models used to analyze potential associations between sealant retention and relevant co-variants.

Loss of Retention Hazard Ratio (95% CI)	Cumulative Retention—3-Year Follow-Up
Age <11 years	1
Age ≥11 years	0.87 (0.51–1.47) *p* = 0.60
Sex—Female	1
Sex—Male	0.69 (0.39–1.21) *p* = 0.20
Study center—University	1
Study center—Dental practice	0.73 (0.19–2.72) *p* = 0.64
Physician—JK & PG	1
Physician—HS	7.10 (2.13–23.81) *p* = 0.001
Test sealants	1
Control sealants	0.82 (0.50–1.35) *p* = 0.44

## Data Availability

The datasets used and/or analyzed during the study are available from the corresponding author upon reasonable request.
